# Clinical Effect of Bone Filling Mesh Container Vertebroplasty in Osteoporotic Compression Fracture

**DOI:** 10.1155/2022/5029679

**Published:** 2022-08-02

**Authors:** Zhiqian Wang, Guomin Li, Jingming He, Haifeng Huang, Anju Zhao

**Affiliations:** ^1^Department of Orthopedics, Guizhou Provincial People's Hospital, Guiyang 550002, China; ^2^Department of Cardres, Guizhou Provincial People's Hospital, Guiyang 550002, China

## Abstract

**Objective:**

To investigate the clinical application of bone filling mesh container vertebroplasty in osteoporotic vertebral compression fractures (OVCFs).

**Methods:**

Patients with OVCF from October 2018 to April 2020 were selected. Patients in the control and study groups underwent percutaneous kyphoplasty (PKP) and bone filling mesh container vertebroplasty, respectively. The Oswestry Disability Index (ODI), Japanese Orthopaedic Association (JOA), visual analog scale (VAS) scores before and after surgery, and the incidence of complications were compared between the two groups.

**Results:**

The operation time and fluoroscopy time of the study group were significantly lower than those of the control group (*P* < 0.05). There was no significant difference in the injection volume of bone cement between the study group and the control group (*P* > 0.05). There was no significant difference in Cobb angle between the two groups. Three months after the operation, the height of the anterior edge increased and the Cobb angle decreased in the two groups (*P* < 0.05), but there was no significant difference in the height of the anterior edge and the Cobb angle between the two groups (*P* > 0.05). The JOA scores increased, while the ODI and VAS scores decreased in both groups after surgery (*P* < 0.05). There was no significant difference in the total effective rate between the study group (96.15%) and the control group (92.31%) (*P* > 0.05). The incidence of complications in the study group (3.85%) was significantly lower than that in the control group (15.38%) (*P* < 0.05).

**Conclusions:**

For the treatment of OVCFs, bone filling mesh container vertebroplasty is comparable to PKP in terms of functional recovery, but it can safely reduce operative time, fluoroscopy time, and complication rates.

## 1. Introduction

Osteoporotic vertebral compression fractures (OVCFs) are the most common diseases in orthopaedics, mainly due to wedge-shaped compressive deformation caused by the axial force mechanism. In recent years, with the intensification of population aging, the incidence of OVFC has been increasing, which seriously threatens the quality of life of the aging population [1–3].

Surgical operation is the treatment of choice for OVCFs, and percutaneous kyphoplasty (PKP) is the most common type which mainly involves injecting bone cement into the vertebral body to restore the stiffness and strength of the vertebral body and relieve pain. However, in these patients, the posterior wall of the PKP is fractured, and the vertebral canal is connected to the vertebral body. Therefore, when PKP is implemented, bone cement leaks into the vertebral canal along the damaged part of the posterior wall of the vertebral body, causing spinal cord and nerve injury and making it difficult to achieve satisfactory results [4, 5]. Bone filling mesh container vertebroplasty is a novel operation technique based on PKP, which can minimize the incidence of bone cement leakage and ensure effectiveness and safety of treatment [6, 7].

The purpose of this study was to explore and determine the clinical value of bone filling mesh container vertebroplasty. Our results suggested that for the treatment of OVCFs, bone filling mesh container vertebroplasty is comparable to PKP in terms of functional recovery, reducing operative time, number of fluoroscopy, and complication rates.

## 2. Materials and Methods

### 2.1. Baseline Data

This study was approved by the Ethics Committee of our hospital. A total of 104 patients with osteoporotic compression fracture in our hospital from October 2018 to April 2020 were selected and divided into the study and control groups using a simple randomized method; each group had 52 cases. In the control group, there were 24 males and 28 females, ranging in age from 56 to 79 years, with an average age of 67.56 ± 10.79 years. Fracture segments are as follows: T_11_, 10 cases; T_12_, 19 cases; L_1_, 12 cases; and L_2_, 11 cases. In the study group, there were 22 males and 30 females, ranging in age from 54 to 78 years, with an average age of 69.01 ± 9.33 years. Fracture segments are as follows: T_11_, 7 cases; T_12_, 21 cases; L_1_, 14 cases; and L_2_, 10 cases; clinical data such as age, sex, and fracture segments were not significantly different between the two groups (*P* > 0.05).

### 2.2. Selection Criteria

The inclusion criteria for this study were (1) low signal on T1-weighted MR image, medium-high signal on T2-weighted image, and fat-suppressed image; (2) BMD T value ≤ −2.5; and (3) first fracture. The exclusion criteria were (1) pathological fracture caused by vertebral tumor, (2) coagulation disorder, (3) allergic constitution, (4) spinal cord and nerve compression, (5) mental system disease, and (6) not fully tolerated surgeon.

### 2.3. Interventions

For the patients in the study group, bone filling mesh container vertebroplasty was adopted, patients were assisted to take the prone position, and the chest and iliac front were padded; thereafter, general anesthesia was administered. After C-arm assisted fluoroscopic positioning, the needle was inserted at 2:00 on the right side and 10:00 on the left side. The inner core was extracted, and a guide wire was inserted. The guide wire needle path was expanded by fine drilling, and the bone dilation orthosis was used to make it approximately 1/3 of the position before the midline of the diseased vertebra. The bone dilation orthosis was opened. After satisfactory reduction of the vertebral body, the bone expansion orthosis was removed, and a bone-filled net bag of appropriate specifications was placed in front of approximately 1/4 of the affected vertebral body. The bone cement was perfused slowly and repeatedly at a low pressure. Perfusion was terminated when the filling mesh container was fully expanded in the vertebral body/bone to prevent cement leakage.

For the patients in the control group, PKP was adopted. The methods of bone cement perfusion, puncture, surgical position, and anesthesia were the same as those used in the study group. The working casing pipe was replaced after successful puncture, and then the guide needle, dilator tube, and working casing pipe were inserted step-by-step to build the working channel which was placed in approximately 1/3 of the anterior part of the midline of the affected vertebrae; iohexol was injected to expand the saccule, which was then removed after satisfactory reduction of the vertebral body, and bone cement was injected. Perfusion was terminated when there was leakage or even dispersion of the bone cement.

### 2.4. Observation Indices

(1) The operation conditions including operation time, perspective times, and bone cement injection volume were counted. (2) The scores of thoracolumbar function and pain before and after surgery in the two groups were statistically analyzed. The thoracolumbar function was evaluated according to the Oswestry Disability Index (ODI) and Japanese Orthopaedic Association (JOA) assessment treatment. The JOA score ranges from 0 to 29, the higher the score, the better the function of the thoracolumbar spine; the ODI score ranges from 0 to 50, the higher the score, the more serious the dysfunction, and the pain score was evaluated by the visual analog scale (VAS), the higher the score, the stronger the pain [8]. (3) X-ray films of the diseased vertebral bodies were obtained at different points in time. The height of the anterior border and Cobb angle in the two groups pre- and postoperative were measured. (4) The treatment effects in the two groups were assessed. No improvement in the Cobb angle, anterior femoral, and lumbar bone density, and low back pain was regarded as invalidation. When the Cobb angle recovered to some extent, the bone density of the femoral front and lumbar vertebrae increased by 0%–2%, and the symptoms of low back pain were relieved to some extent, but slight pain still persisted, and it was regarded as effective; when the Cobb angle recovered significantly, the bone mineral density of the front end of the femur and lumbar spine increased by more than 2%, and the symptoms of back pain disappeared; it was regarded as remarkable effectiveness: (remarkable effectiveness + effectiveness)/total cases × 100% = total effective rate [9]. (5) Complication rates in the two groups were assessed.

### 2.5. Statistical Analysis

SPSS 22.0 (IBM, USA) was used for data analysis. Measurement data were expressed as mean±SD. Enumeration data were expressed as *n* (%), *χ*^2^ test. Statistical significance was set at *P* < 0.05.

## 3. Results

### 3.1. The Operation Condition

As shown in Table 1, the operation time of the study group (34.64 ± 6.78 min) was significantly lower than that of the control group (40.31 ± 9.69 min). The number of fluoroscopies in the study group (9.22 ± 1.98 times) was significantly lower than that in the control group (12.89 ± 3.64 times) (*P* < 0.05). The injection volume of bone cement in the study group was 5.01 ± 0.98 ml, which was not significantly different from that in the control group (5.11 ± 1.05 ml) (*P* > 0.05).

### 3.2. The Height of the Anterior Border and Cobb Angle

Preoperatively, in the study group, the height of the anterior border was 31.15 ± 6.29% and Cobb angle was 22.18 ± 5.94°, while in the control group, the height of the anterior border was 30.03 ± 7.10% and the Cobb angle was 21.39 ± 6.44°, and there was no significant difference between the two groups (*P* > 0.05). Three months postoperative, the height of the anterior borders in the two groups increased, while the Cobb angles decreased (*P* < 0.05). However, in the study group, the height of the anterior border was 70.78 ± 5.34% and Cobb angle 9.67 ± 2.38°, while in the control group, the height of the anterior border was 71.78 ± 4.89% and Cobb angle 10.25 ± 2.56°, and there was no significant difference between the two groups (*P* > 0.05, Table 2).

### 3.3. The ODI, JOA, and VAS Scores

Preoperatively, the ODI, JOA, and VAS scores in the study group were 45.78 ± 4.69, 13.10 ± 3.78, and 7.78 ± 1.14 points, respectively, while those in the control group were 46.39 ± 5.11, 12.99 ± 4.01, and 8.01 ± 1.51 points, respectively; there was no significant difference between the two groups (*P* > 0.05). Postoperatively, the JOA score in the two groups increased, while the ODI and VAS scores decreased (*P* <0.05). However, the ODI, JOA, and VAS scores in the study group were 9.09 ± 1.18, 27.33 ± 3.29, and 1.89 ± 0.59, respectively, while those in the control group were 8.96 ± 1.39, 28.19 ± 3.56, and 2.01 ± 0.62 points, respectively, and there was no significant difference between the two groups (*P* > 0.05, Table 3).

### 3.4. The Treatment Effect

As shown in Table 4, there was no significant difference in the total effective rate between the study group (96.15%) and the control group (92.31%) (*P* > 0.05).

### 3.5. The Complications

The complication rate in the study group (3.85%) was significantly lower than that in the control group (15.38%) (*P* < 0.05, Table 5).

## 4. Discussion

OVCFs cause severe low back pain, changes in the physiological curvature of the spine, and limitation of activities, which greatly affects the quality of life of patients [10, 11]. The conservative treatment cycle of OVCFs is long, the patient's compliance with treatment is poor, and it is unbearable to stay in bed for a long time [12]. Therefore, surgical operation remains the treatment of choice for OVCFs.

PKP is an important minimally invasive surgery for the clinical treatment of OVCFs. With the aid of imaging technology, PKP can improve the accuracy of locating the fracture site and condition, thereby improving the effect of surgical treatment [13]. PKP is mainly used for percutaneous injection of bone cement to improve the stability and strength of the injured vertebra and restore the angle and height of the intervertebral space [14]. However, when the saccule was opened during PKP, the fracture block moved to the spinal canal. When the bone cement was perfused, it could enter the spinal canal along the ruptured site of the posterior wall of the vertebral body, resulting in compression of the spinal nerves, thus affecting the safety of treatment [15, 16]. According to Bozzo and Bhandari [17], PKP can effectively restore the height of the vertebral body after balloon dilation. A cavity can be formed in the vertebral body, which is conducive to the injection and diffusion of bone cement. However, after the balloon is opened, the balloon needs to be pulled out and then injected with bone cement, which is likely to cause the vertebral body to rebound, resulting in the loss of the height of the vertebral body, and it is difficult for the vertebral body to form a cavity. If the bone cement is injected again, leakage will occur. The bone-filled mesh container is a new type of material, which has the advantages of good ductility and strong compressibility. It has been widely used in OVCF. In vertebroplasty, a bone filling mesh container is positioned and placed in an open channel, and bone cement is injected into the mesh container to restore the height of the affected vertebroplasty through its wrapping action. With its strong shear resistance, it reduces the risk of complications and ensures the effectiveness and safety of the treatment [18].

In this study, there was no significant difference in the amount of bone cement injected between the study group and the control group, but the operation time of the study group was shorter than that of the control group, and the number of fluoroscopy was less than that of the control group. This is mainly because PKP is performed with bone cement injection after the balloon is opened and deflated, while bone filling mesh container vertebroplasty is performed at the same time as bone cement injection, so it is beneficial to shorten the operation time and the frequency of intraoperative radiation exposure. In addition, postoperative anterior edge height increased, Cobb angle decreased, JOA score increased, and ODI and VAS scores decreased in both groups. However, the difference between the two groups was not significant, indicating that the treatment of bone filling mesh container vertebroplasty in OVCFs was similar to that in PKP. The reason may be that in bone filling mesh container vertebroplasty, the heat generated by the polymerization of the bone cement can damage the sensory nerve endings of the damaged vertebral body, thereby reducing pain. Moreover, the structure of the bone filling mesh container can ensure the effective injection and diffusion of bone cement, strengthen the combination with the surrounding bone tissue, obtain good vertebral body stability, and ensure the improvement of thoracolumbar vertebra function. Several clinical studies have shown that bone filling mesh container vertebroplasty involves inserting a mesh container into the fractured vertebral body through a puncture channel and then injecting bone cement into the mesh container. The “onion effect” occurs when hydrostatic pressure increases the pressure inside the capsule, decreasing from the center to the outer layers to fill the vertebral bodies and lift the endplates. And the mesh container has holes in its surface through which the bone cement can seep out under pressure, anchoring and securing it to the surrounding bone. Simultaneous opening and filling of the mesh bag in the vertebral body prevents correction of kyphosis and loss of height [19, 20]. In addition, complications are an important factor affecting the treatment effect of OVCFs and the functional recovery of vertebral body, among which bone cement leakage is the most common. However, our study confirmed that the incidence of complications in the study group was lower than that in the control group, suggesting that bone filling mesh container vertebroplasty can not only achieve the same therapeutic effect as PKP in patients with OVCFS but also greatly reduce the incidence of bone cement leakage risk and ensure the safety of treatment. The main reason is that in the bone-filled mesh container vertebroplasty, after infusion, the bone cement can be gradually dispersed into the space between the trabecular bone in the vertebral body through the mesh in the capsule to ensure the stability of the bone cement and the bone tissue and reduce the risk of bone cement leakage.

## 5. Conclusion

For the treatment of OVCFs, bone filling mesh-vessel vertebroplasty is comparable to PKP in terms of functional recovery, reducing operative time, number of fluoroscopy, and complication rates.

## Figures and Tables

**Table 1:** Comparison of operation conditions between the two groups ($\bar{x}\pm s$).

Group	Cases	Operation time (min)	Perspective times (times)	Bone cement injection volume (ml)
Study	52	34.64 ± 6.78	9.22 ± 1.98	5.01 ± 0.98
Control	52	40.31 ± 9.69	12.89 ± 3.64	5.11 ± 1.05
*t* value		3.457	6.384	0.502
*P* value		0.001	0.001	0.617

**Table 2:** Comparison of the height of the anterior border and Cobb angle between the two groups ($\bar{x}\pm s$).

Time	Group	Cases	The height of the anterior border (%)	Cobb angle (°)
Before the operation	Study	52	31.15 ± 6.29	22.18 ± 5.94
	Control	52	30.03 ± 7.10	21.39 ± 6.44
	*t* value		0.851	0.650
	*P* value		0.397	0.517
Three months after the operation	Study	52	70.78 ± 5.34^a^	9.67 ± 2.38^a^
	Control	52	71.78 ± 4.89^a^	10.25 ± 2.56^a^
	*t* value		0.996	1.197
	*P* value		0.322	0.234

Note: compared with preoperative values, ^a^*P* < 0.05.

**Table 3:** Comparison of the ODI, JOA, and VAS scores between the two groups ($\bar{x}\pm s$, points).

Time	Group	Cases	ODI	JOA	VAS
Before the operation	Study	52	45.78 ± 4.69	13.10 ± 3.78	7.78 ± 1.14
	Control	52	46.39 ± 5.11	12.99 ± 4.01	8.01 ± 1.51
	*t* value		0.634	0.144	0.877
	*P* value		0.527	0.886	0.383
Six months after the operation	Study	52	9.09 ± 1.18^a^	27.33 ± 3.29^a^	1.89 ± 0.59^a^
	Control group	52	8.96 ± 1.39^a^	28.19 ± 3.56^a^	2.01 ± 0.62^a^
	*t* value		0.514	1.279	1.011
	*P* value		0.608	0.204	0.314

Note: compared with preoperative values, ^a^*P* < 0.05. ODI: Oswestry Disability Index; JOA: Japanese Orthopaedic Association; VAS: visual analog scale.

**Table 4:** Comparison of the effects of treatment between the two groups (*n* (%)).

Group	Cases	Remarkable effectiveness	Effectiveness	Invalidation	Total effective rate
Study	52	34 (65.38)	16 (30.77)	2 (3.85)	50 (96.15)
Control	52	29 (55.77)	19 (36.54)	4 (7.69)	48 (92.31)
*χ* ^2^ value					0.177
*P* value					0.674

**Table 5:** Comparison of the complication rate between the two groups (*n* (%)).

Group	Cases	Bone cement leakage	Deep vein thrombosis in lower limbs	Infection	Vascular injury	The total incidence
Study	52	1 (1.92)	0 (0.00)	0 (0.00)	1 (1.92)	2 (3.85)
Control	52	5 (9.62)	1 (1.92)	1 (1.92)	1 (1.92)	8 (15.38)
*χ* ^2^ value						3.983
*P* value						0.046

## Data Availability

The authors confirm that the data supporting the findings of this study are available within the article.

## References

[1] Chandra R. V., Maingard J., Asadi H. (2018). Vertebroplasty and kyphoplasty for osteoporotic vertebral fractures: what are the latest data. *AJNR. American Journal of Neuroradiology*.

[2] Li H. M., Zhang R. J., Gao H. (2018). New vertebral fractures after osteoporotic vertebral compression fracture between balloon kyphoplasty and nonsurgical treatment PRISMA. *Medicine*.

[3] Buchbinder R., Johnston R. V., Rischin K. J. (2018). Percutaneous vertebroplasty for osteoporotic vertebral compression fracture. *Cochrane Database of Systematic Reviews*.

[4] Wang B., Zhao C. P., Song L. X., Zhu L. (2018). Balloon kyphoplasty versus percutaneous vertebroplasty for osteoporotic vertebral compression fracture: a meta-analysis and systematic review. *Journal of Orthopaedic Surgery and Research*.

[5] Musbahi O., Ali A. M., Hassany H., Mobasheri R. (2018). Vertebral compression fractures. *British Journal of Hospital Medicine*.

[6] Parreira P., Maher C. G., Megale R. Z., March L., Ferreira M. L. (2017). An overview of clinical guidelines for the management of vertebral compression fracture: a systematic review. *The Spine Journal*.

[7] Lamanna A., Maingard J., Kok H. K. (2019). Vertebroplasty for acute painful osteoporotic vertebral compression fractures: an update. *Journal of Medical Imaging and Radiation Oncology*.

[8] Hinde K., Maingard J., Hirsch J. A., Phan K., Asadi H., Chandra R. V. (2020). Mortality outcomes of vertebral augmentation (vertebroplasty and/or balloon kyphoplasty) for osteoporotic vertebral compression fractures: a systematic review and meta-analysis. *Radiology*.

[9] Zhu Y., Cheng J., Yin J., Zhang Z., Liu C., Hao D. (2019). Therapeutic effect of kyphoplasty and balloon vertebroplasty on osteoporotic vertebral compression fracture: a systematic review and meta-analysis of randomized controlled trials. *Medicine*.

[10] Hoyt D., Urits I., Orhurhu V. (2020). Current concepts in the management of vertebral compression fractures. *Current Pain and Headache Reports*.

[11] Zhang H., Xu C., Zhang T., Gao Z., Zhang T. (2017). Does percutaneous vertebroplasty or balloon kyphoplasty for osteoporotic vertebral compression fractures increase the incidence of new vertebral fractures? A meta-analysis. *Pain Physician*.

[12] Yuan W. H., Hsu H. C., Lai K. L. (2016). Vertebroplasty and balloon kyphoplasty versus conservative treatment for osteoporotic vertebral compression fractures: a meta-analysis. *Medicine*.

[13] Zuo X. H., Zhu X. P., Bao H. G. (2018). Network meta-analysis of percutaneous vertebroplasty, percutaneous kyphoplasty, nerve block, and conservative treatment for nonsurgery options of acute/subacute and chronic osteoporotic vertebral compression fractures (OVCFs) in short-term and long-term effects. *Medicine*.

[14] Ebeling P. R., Akesson K., Bauer D. C. (2019). The efficacy and safety of vertebral augmentation: a second ASBMR task force report. *Journal of Bone and Mineral Research*.

[15] Noriega D., Marcia S., Theumann N. (2019). A prospective, international, randomized, noninferiority study comparing an implantable titanium vertebral augmentation device versus balloon kyphoplasty in the reduction of vertebral compression fractures (SAKOS study). *The Spine Journal*.

[16] Kim H. S., Ju C. I. (2017). Spinal instability predictive scoring system for subsequent fracture after bone cement augmentation in patients with osteoporotic vertebral compression fracture. *World Neurosurgery*.

[17] Bozzo A., Bhandari M. (2018). Cochrane in CORR?: percutaneous vertebroplasty for osteoporotic vertebral compression fracture. *Clinical Orthopaedics and Related Research*.

[18] Xu J., Lin J., Li J., Yang Y., Fei Q. (2019). "Targeted percutaneous vertebroplasty" versus traditional percutaneous vertebroplasty for osteoporotic vertebral compression fracture. *Surgical Innovation*.

[19] Jacobs E., Senden R., McCrum C., van Rhijn L. W., Meijer K., Willems P. C. (2019). Effect of a semirigid thoracolumbar orthosis on gait and sagittal alignment in patients with an osteoporotic vertebral compression fracture. *Clinical Interventions in Aging*.

[20] McCarthy J., Davis A. (2016). Diagnosis and management of vertebral compression fractures. *American Family Physician*.

